# Pain trajectory defines knee osteoarthritis subgroups: a prospective observational study

**DOI:** 10.1097/j.pain.0000000000001975

**Published:** 2020-06-24

**Authors:** Maja R. Radojčić, Nigel K. Arden, Xiaotian Yang, Victoria Y. Strauss, Fraser Birrell, Cyrus Cooper, Stefan Kluzek

**Affiliations:** a Nuffield Department of Orthopaedics, Rheumatology and Musculoskeletal Sciences, University of Oxford, Oxford, United Kingdom; b Centre for Sport, Exercise and Osteoarthritis Research Versus Arthritis, University of Oxford, Oxford, United Kingdom; c Department of Rehabilitation Medicine, West China Hospital, Sichuan University, Chengdu, China; d Nuffield Department of Orthopaedics, Rheumatology and Musculoskeletal Sciences, Centre for Statistics in Medicine, University of Oxford, Oxford, United Kingdom; e MRC-Versus Arthritis Centre for Integrated Research Into Musculoskeletal Ageing, University of Newcastle, Newcastle upon Tyne, United Kingdom; f MRC Lifecourse Epidemiology Unit, University of Southampton, Southampton, United Kingdom; g Department of Sports Medicine, University of Nottingham, Nottingham, United Kingdom

**Keywords:** Pain, Osteoarthritis, Group-based trajectory modelling, Phenotype

## Abstract

Knee osteoarthritis (OA) is a heterogeneous disease, and identification of its subgroups/phenotypes can improve patient treatment and drug development. We aimed to identify homogeneous OA subgroups/phenotypes using pain development over time; to understand the interplay between pain and functional limitation in time course; and to investigate subgroups' responses to available pharmacological and surgical treatments. We used group-based trajectory modelling to identify pain trajectories in the phase-3 VIDEO trial (n = 474, 3-year follow-up) and also in the Osteoarthritis Initiative cohort study (n = 4796, 9-year follow-up). We extended trajectory models by (1) fitting dual trajectories to investigate the interplay between pain and functional limitation over time, and (2) including analgesic use as a time-varying covariate. Also, we investigated the relationship between trajectory groups and knee replacement in regression models. We identified 4 pain trajectory groups in the trial and 6 in the cohort. These overlapped and led us to define 4 OA phenotypes: low-fluctuating, mild-increasing, moderate-treatment-sensitive, and severe-treatment-insensitive pain. Over time, functional knee limitation followed the same trajectory as pain with almost complete concordance (94.3%) between pain and functional limitation trajectory groups. Notably, we identified a phenotype with severe pain that did not benefit from available treatments, and another one most likely to benefit from knee replacement. Thus, knee OA subgroups/phenotypes can be identified based on patients' pain experiences in studies with long and regular follow-up. We provided a robust approach, reproducible between different study designs, which informs clinicians about symptom development and delivery of treatment options and opens a new avenue toward personalized medicine in OA.

## Abstract 2

Osteoarthritis subgroups/phenotypes, based on pain experience over time, inform on symptom development and delivery of treatment options and open a new avenue toward personalized medicine.

## 1. Introduction

Pain is the primary symptom and descriptor of the burden of osteoarthritis (OA), a chronic disease related to substantial disability, morbidity, and costs.^29^ According to 2010 estimates, it is globally among the top contributors to disability.^7,22^ The socioeconomic burden of OA includes direct expenditure on nonpharmacological and pharmacological treatments, with indirect costs from productivity loss, early retirement, and premature death.^13^ For individuals, long-term outcomes include pain, functional limitations of the affected joint, and reduced quality of life. Currently available treatment options—both pharmacological for pain/symptom management and surgical, ie, joint replacement—do not provide significant improvements to all patients.^30,31^

The need for successful development of treatment options for all OA patients is currently unmet. Although treatment is expected to halt or minimise OA progression, pain relief is the essential determinant of cost-effectiveness.^8^ There have been considerable efforts to develop OA treatment, with many promising candidates failing to reach endpoints in phase-3 clinical studies. This challenge has been explained by heterogeneity, indicating that OA requires personalised medicine.^12,17,28^ Thus, to improve drug development and reduce OA burden, proper identification of phenotypes—distinct groups of patients that share the same pathophysiology—is required.^21^ These phenotypes will help in selecting patients most likely to benefit from specific treatment options.

In this study, we hypothesised that OA phenotypes could be identified by patients' pain/symptom experiences over time. We focused on the most common one: knee OA.^12^ We used a phase-3 clinical trial as a typically selected clinical OA population, and a more extensive prospective cohort study for the external validation. We intended to identify pain patterns/trajectories and to explore the interplay between pain and functional limitation development over time because both are outcomes of interest. To identify if and how phenotypes respond to available pharmacological treatments, we investigated the effect of medication over time. We also studied whether pain trajectory groups are associated with surgical outcomes. Finally, we explored the baseline characteristics associated with each phenotype because these could provide evidence-based recommendations for core phenotyping in personalised medicine and trial recruitment.

## 2. Methods

### 2.1. Study samples

The Vitamin D Effect on Osteoarthritis (VIDEO) trial was designed to investigate the effect of vitamin D supplementation (daily 800 IU oral cholecalciferol) on knee OA progression. It was a multicentre, 3-year, double-blind, placebo-controlled randomised clinical trial approved, registered, and performed in the United Kingdom (EudraCT: ref.2004-000169-37, ISRCTN94818153, CTA No.11287/0001/001).^2^ Participants were included if older than 50 years with radiological evidence of knee OA and knee pain for most days of the month. Exclusion criteria were: morning knee stiffness longer than 30 minutes, secondary or inflammatory arthritis, history of knee surgery or knee replacement in previous 6 months, osteoporotic fractures, and use of bisphosphonates, supplements containing vitamin D, and glucosamine and chondroitin less than 3 months. For further details, see the study by Arden et al.^2^ Although an interventional study, for this work, the VIDEO trial was used as an observational typically selected clinical OA study sample.

The Osteoarthritis Initiative (OAI) study is a prospective observational study of knee OA sponsored by the National Institute of Health. Participants age 45 to 79 years were recruited at 4 centres across the United States. Exclusion criteria were: inflammatory arthritis, severe joint space narrowing, bilateral knee replacement or plans for it in the next 3 years, comorbidities that might interfere with participation in this study, participation in clinical trials, and others. Additional study details, as well as data, are available at the https://nda.nih.gov/oai. As of February 2019, data were available through the ninth-year visit.

Participants in both studies provided written informed consents.

### 2.2. Outcome measures

The primary outcome in this study was the pain subscale of the Western Ontario and McMaster Universities Osteoarthritis Index (WOMAC)^5^—a total score of 5 questions scaled from zero (no pain) to 100 (extreme pain). In the VIDEO trial, pain was assessed for the index knee as previous 48 hour-recall at 6-month intervals (7 repeated measures). In the OAI study, the timeframe of pain assessment was previous 7 days at annual intervals (10 repeated measures). There were reports for the left knee and right knee irrespective of the disease. We assigned a more painful knee throughout the visits for result generalisation.

The secondary outcomes in this study were the functional limitation subscale of the WOMAC,^5^ assessed and scaled like the pain subscale (0-100), and surgical outcome, ie, knee replacement. In the VIDEO trial, knee replacement was recorded at the end of the trial as binary outcome. In the OAI study, exact dates of the knee replacements were recorded throughout the follow-up.

### 2.3. Covariates

Baseline variables were used for descriptive purpose and to assess their impact on the pain, as well as confounding variables when the pain was related to the surgical outcome. Age, sex, smoking, alcohol use, employment status, and use of supplements, glucosamine, and chondroitin were self-reported. Body mass index (BMI) was computed based on height and weight measurements. In the VIDEO trial, depression was assessed by Beck Depression Inventory containing 21 questions summed to the total score (0-63).^4^ In the OAI study, Centre for Epidemiological Studies Depression Scale with 20 items and a total score 0 to 60 was used.^20^ In both questionnaires, a higher score indicates worse symptomatology. In the VIDEO trial only, physical activity (once or more times per month) and quality of life were assessed. The short version of the World Health Organization Quality of Life (WHOQoL-Bref) contains 26 questions measuring 4 domains, physical health, psychological health, social relationships, and environment, each scoring from 0 to 100. Higher scores denote a better quality of life.^10^ Comorbidities, defined as none, one, or more than one, were recorded in the OAI study only. A trained orthopaedic fellow or radiologist scored the radiographs according to the Kellgren–Lawrence (KL) grades.^18^

Use of currently available medications that affect pain levels, directly or indirectly, ie, analgesics, nonsteroid anti-inflammatory drugs, and steroids, further referred as analgesics, was recorded in both studies throughout follow-up and used in the primary analysis as a binary time-varying covariate. Mortality during follow-up was used for descriptive purposes and sensitivity analyses. Missing values were shown per variable; these were not imputed and were considered for analyses if the percentage was less than 10%.

### 2.4. Statistical analysis

First, we showed baseline characteristics of our study samples for descriptive purposes. Furthermore, we conducted our analyses in 3 steps: identification of trajectories with 2 extensions, investigating the association of pain trajectories with distal surgical outcome, and identification of baseline factors for predicting trajectories.

To identify trajectory groups (latent clusters of individuals) that follow a similar pattern of how pain develops over time, we used group-based trajectory modelling.^23^ We used censored normal models with up to a fourth-order polynomial and tested a different number of trajectory groups. Statistical criteria, Bayesian information criteria,^15^ and group posterior probability (>0.70)^24^ aided in selecting the best model fit.^1,16^ We also used the Wald test for equality of trajectory coefficient estimates to confirm that trajectories are distinctive or parallel.^14^ Our trajectories were related to the index knee in the VIDEO trial, and more painful knee in the OAI study. After fitting pain trajectories, we included 2 model extensions, dual trajectories and time-varying covariate.^14^ Dual trajectory modelling is analysing the developmental course of 2 different but related outcomes.^25^ Here, we looked into pain and functional limitations. We modelled functional limitation trajectories in the same manner as pain trajectories. Then, in the dual trajectory model, we examined pain development over time, given the information from function limitation trajectories. This analysis provides conditional probabilities joining membership across the pain and functional limitation trajectory groups.^14^ In the last section of trajectory modelling, we included analgesic use as time-varying covariates into pain trajectory model.^14^ It is a binary variable because the purpose of this subanalysis was to find whether currently available analgesics significantly reduced pain over time providing the effect estimates per trajectory groups (the strata of indication severity).

To investigate the association between pain trajectory groups and knee replacement, in the VIDEO trial, we used a logistic regression forward selection method. In the OAI study, we conducted a time-to-event analysis using the Cox proportional-hazards forward selection model.

To identify baseline factors associated with pain trajectory membership and to differentiate each trajectory group, we used a multinomial regression forward selection method. We created several models with different trajectory groups of interest as referenced ones.

As sensitivity analyses, we remodelled pain trajectories excluding mortality cases during the follow-up. Furthermore, in the OAI study, we investigated the left and right knee pain trajectories. Also, dual left and right trajectories, ie, modelling left knee pain development over time having the right knee pain trajectories.

We analysed the data using SAS 9.4 (SAS Institute, Cary, NC). We used proc traj package with macros trajtest and trajplotnew available at https://www.andrew.cmu.edu/user/bjones/.

## 3. Results

The VIDEO trial included 474 participants, whereas the OAI study had 4796. Table 1 contains baseline characteristics of the study samples. Descriptive statistics, including missing values of pain, functional limitation, and analgesic use variables at every follow-up visit used for the trajectory modelling, are included in Appendix (available as supplemental digital content at http://links.lww.com/PAIN/B88).

**Table 1:** Baseline descriptive statistics of the study samples.

Variable	The VIDEO trial (N = 474) follow-up 3 years			The OAI study (N = 4796) follow-up 9 years		
	N	%	Mean (SD)/median (IQR)	N	%	Mean (SD)/median (IQR)
Treatment				N/A		
Active	237	50.0				
Vitamin D				N/A		
Active	232		22.9 (8.8)/21.9 (16.3-28.3)			
Placebo	231		23.0 (8.0)/22.3 (16.7-28.8)			
Missing	11	2.3				
Age	474		64.0 (7.6)/63.0 (58.0-69.0)	4500		61.3 (9.2)/61.0 (53.0-69.0)
Missing	0			296	6.2	
Sex						
Women	289	61.0		2804	58.5	
BMI	473		29.4 (5.1)/28.7 (25.5-32.3)	4792		28.6 (4.8)/28.3 (25.1-31.7)
Missing	1	0.2		4	0.1	
Smoking						
Current	25	5.2		313	6.5	
Current-not regular				10	0.2	
Former	214	45.2		1909	39.8	
Never smoked	230	48.5		2564	53.5	
Missing	5	1.1		0		
Alcohol use						
Yes	395	83.3		3821	79.7	
Missing	0			0		
Currently working						
Yes	198	41.8		2943	61.4	
Missing	1	0.2		0		
Physical activity sport/hobby>1/month				N/A		
Yes	224	47.3				
Missing	4	0.8				
Depression						
Beck Depression Inventory (score 0-63)	473		2.0 (2.6)/1.0 (0.0-3.0)			
Centre for Epidemiological Studies Depression Scale (score 0-60)				4731		6.6 (7.0)/4.0 (2.0-9.0)
Missing	1	0.2		65	1.4	
Quality of life WHOQoL-Bref (score 0-100)					N/A	
Physical domain	468					
Psychological domain	468		64.5 (16.6)/64.3 (53.6-75.0)			
Social domain	468		71.2 (14.3)/70.8 (62.5-79.2)			
Environmental domain	468		71.9 (18.8)/75.0 (58.3-83.3)			
Missing	6	1.3	77.4 (13.0)/78.1 (68.8-87.5)			
Comorbidities	N/A					
None				3631	75.7	
One				724	15.1	
More than one				441	9.2	
Missing				0		
Medications use of analgesics, NSAIDs, and steroids						
Yes	273	57.6		1783	37.2	
Missing	17	3.6		0		
Supplements use of glucosamine and chondroitin						
Yes	139	29.3		1625	33.9	
Missing	17	3.6		0		
Kellgren–Lawrence grade (Index/Worse knee)*						
0	6	1.3		1260	26.3	
1	121	25.5		697	14.5	
2	178	37.6		1365	28.5	
3	136	28.7		892	18.6	
4	29	6.1		293	6.1	
Missing	4	0.8		289	6.0	
Knee replacement at baseline						
Yes				63	1.3	
Left				25	0.5	
Right				38	0.8	
No	474	100.0		4733	98.7	
Missing	0			0		
Knee replacement during follow-up						
Left				271	5.7	
Right				277	5.8	
Bilateral replacement	2	0.4		119	2.5	
Index	13	2.7				
Contralateral	30	6.3				
Individuals with knee replacement by the end of the study†						
Yes	41	8.6		492	9.2	
No	433	91.4		4357	90.8	
Mortality during follow-up						
Yes	5	1.1		305	6.4	

* The index knee refers to the VIDEO trial, and the Worse knee to the OAI study.

† The summary of the previous 2 variables, knee replacement at baseline and during follow-up, showing information per person instead of per knee.

BMI, body mass index; IQR, interquartile range; N/A, not applicable or not assessed; NSAID, nonsteroidal anti-inflammatory drug; OAI, Osteoarthritis Initiative; WHOQoL-Bref, The World Health Organization Quality of Life Instrument.

### 3.1. Trajectories

**Figure 1.**
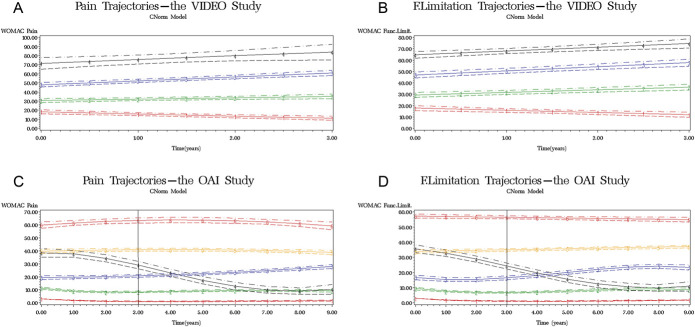
Trajectories—red colour (-1-) indicates the first trajectory group, green (-2-) the second, blue (-3-) the third, black (-4-) the fourth, yellow (-5-) the fifth, and orange (-6-) the sixth group; (A) pain trajectories in the VIDEO trial; (B) functional limitation trajectories in the VIDEO trial; (C) pain trajectories in the OAI study with a window of 3-year follow-up comparable to the VIDEO trial duration; people with minimal-to-neglected knee pain—trajectory 1; low-fluctuating phenotype—trajectory 2 corresponding to the trajectory one in the VIDEO trial; mild-increasing phenotype—trajectory 3 corresponding to the trajectory 2 in the VIDEO trial; moderate-treatment-sensitive phenotype—trajectories 4 and 5 corresponding to the trajectory 3 in the VIDEO trial; high-treatment-insensitive phenotype corresponding to the trajectory 4 in the VIDEO trial; (D) functional limitation trajectories in the OAI study with a window of 3-year follow-up equivalent to the VIDEO trial duration. OAI = Osteoarthritis Initiative.

We identified 4 pain trajectories described by the first-order curves in the VIDEO trial (Fig. 1A, Table 2). The classification of individuals in pain trajectory groups measured by the posterior probability of membership was very good: ranging from 0.85 to 0.91. The Wald test confirmed that the intercepts of all trajectories were statistically, significantly different from each other. The fourth trajectory was parallel (the slope was not statistically different) to the second trajectory (χ^2^ = 3.11, *P* = 0.08) and third trajectory (χ^2^ = 0.01, *P* = 0.93), whereas all others differed. The second trajectory model included dual trajectories. The best model fit identified 4 first-order curves that described functional limitation development (Fig. 1B, modelling details in Appendix, available as supplemental digital content at http://links.lww.com/PAIN/B88). Functional limitation trajectories minimally affected the classification of pain trajectory groups in the dual model (Table 2). Joint probabilities of pain and functional limitation trajectory groups showed that 92.0% of individuals classified in the overlapping groups (Appendix, available as supplemental digital content at http://links.lww.com/PAIN/B88). Finally, in the third trajectory model, analgesic use had a significant positive effect on reducing pain in the first and second trajectory groups and minimally affected the classification of pain groups compared to the primary model (Table 2).

**Table 2:** Pain trajectory modelling.

The main model*					Dual trajectory model†		Model with time-varying covariate‡		
TG	Intercept	Curve order	Group %	Post. prob.	Group %	Post. prob.	Group %	Post. prob.	Covariate estimate (95% CI)
The VIDEO trial									
1	17.6	1	36.5	0.90	31.6	0.93	35.0	0.90	2.6 (0.3 to 4.9)
2	30.5	1	39.7	0.85	36.1	0.88	40.7	0.86	3.1 (0.9 to 5.3)
3	48.0	1	20.5	0.91	20.0	0.88	21.3	0.89	−1.1 (−4.2 to 2.0)
4	71.5	1	3.4	0.89	12.2	0.92	3.0	0.93	−3.6 (−10.3 to 3.1)
The OAI study									
1	−7.4§	3	22.8	0.90	22.9	0.94	22.4	0.88	8.2 (6.9 to 9.5)
2	8.6	4	37.2	0.87	31.6	0.89	39.8	0.85	8.2 (7.6 to 8.9)
3	18.6	2	22.5	0.83	20.6	0.86	21.4	0.80	7.2 (6.3 to 8.1)
4	38.4	3	3.4	0.80	6.8	0.86	10.4	0.83	7.1 (5.8 to 8.3)
5	39.7	2	11.2	0.84	13.4	0.88	3.8	0.87	16.0 (13.6 to 18.5)
6	59.8	2	3.0	0.89	4.6	0.92	2.1	0.79	0.1 (−2.9 to 3.0)

* The model was created using WOMAC pain repeated measures.

† The model was created using WOMAC pain repeated measures given the WOMAC functional limitation trajectories.

‡ The model was created using WOMAC pain repeated measures adjusted for medication use as a time-varying covariate during the follow-up. The model assumption was “no use” at all time points; thus, the covariate estimates are positive and indicate that the use of medication was reducing the pain. In the OAI study only, medication use is missing at follow-up year 3; thus, pain trajectories were redone without year 3, fully reproduced as with year 3 data, and in that model, the time-varying medication use was included. This model did not fully converge. The analysis was redone numerous times, and the estimates were always the same as reported here.

§ The estimate is negative but the actual minimum of the scale is zero.

CI, confidence interval; OAI, Osteoarthritis Initiative; Post. prob., posterior probability; TG, trajectory group.

In the OAI study, we identified 6 trajectories described by higher-order curves: the first and fourth trajectories described by cubic curves, the second one with quartic, and third, fifth, and sixth with quadratic curves (Fig. 1C and Table 2). Posterior probabilities were very good, ranging from 0.80 to 0.90. Intercepts of pain trajectories were different; only group 4 and 5 intercepts were not different (χ^2^ = 0.50, *P* = 0.47). Other parameters were compared between curves of the same order. The cubic curves were not parallel: all parameters were statistically significantly different. The quadratic curves of the fifth and sixth trajectories were parallel (linear component χ^2^ = 2.70, *P* = 0.10; quadratic component χ^2^ = 2.52, *P* = 0.11), whereas the third trajectory was significantly different from these 2. For the second—dual trajectory model—we identified 6 functional limitation trajectories (Fig. 1D, modelling details in Appendix, available as supplemental digital content at http://links.lww.com/PAIN/B88). Additional information on functional limitation development over time introduced slight changes into the pain trajectory groups (Table 2). Joint probabilities of pain and functional limitation development showed that 94.3% of individuals were classified in overlapping groups (Appendix, available as supplemental digital content at http://links.lww.com/PAIN/B88). Finally, for the third trajectory model in this study, we had to remodel pain trajectories without time point 3 because use of analgesics was missing completely (Appendix, available as supplemental digital content at http://links.lww.com/PAIN/B88). The remodelled pain trajectories fully reflected those from the original model, with neglected alterations (Appendix, available as supplemental digital content at http://links.lww.com/PAIN/B88). Analgesics' use had a significant positive effect in all trajectory groups except the sixth and affected the classification of fourth and fifth pain groups.

### 3.2. Risk for the distal outcome

We found that no single pain trajectory group in the VIDEO trial was significantly associated with index knee replacement during the 3-year follow-up. In the OAI study, third, fourth, and fifth groups but not the sixth had significantly higher hazard ratio of having knee replacement during the 9-year follow-up when compared to the second group (Table 3), and all groups when referenced to the first group (Appendix, available as supplemental digital content at http://links.lww.com/PAIN/B88). The highest hazard ratio was in the fourth group.

**Table 3:** Pain trajectories as predictors of knee replacement.

The VIDEO trial		The OAI study			
Pain trajectory	OR* (95% CI) index knee	Pain trajectory	HR† (95% CI) left knee	HR† (95% CI) right knee	HR† (95% CI) generalised
1	Reference	2	Reference		
2	7.0 (0.8-59.0)	3	2.5 (1.8-3.7)	2.4 (1.7-3.5)	1.3 (1.1-1.7)
3	4.8 (0.4-55.9)	4	13.8 (9.4-20.2)	11.9 (8.1-17.6)	4.6 (3.4-6.4)
		5	3.8 (2.4-5.8)	3.5 (2.3-5.3)	1.8 (1.3-2.4)
4	16.6 (0.9-308.6)	6	4.3 (2.0-8.9)	1.8 (0.7-4.6)	1.3 (0.7-2.3)

All models were constructed using forward selection procedure. In the VIDEO trial, variables included in the selection were pain trajectory group, treatment, vitamin D, interaction treatment and vitamin D, age, sex, body mass index, smoking, alcohol drinking, currently working, physical activity, Beck depression scale, physical, psychological, social, and environment domains of the quality of life, Kellgren–Lawrence grade of the index knee at baseline, use of medications, and use of supplements at baseline. In the OAI study, variables included in the model were pain trajectory group, age, sex, body mass index, smoking, alcohol drinking, currently working, Centre for Epidemiological Studies Depression score, comorbidities, Kellgren–Lawrence of the examined knee at baseline, knee replacement at baseline, use of medications, and use of supplements at baseline. The estimates presented were from the final models.

Number of observations used in the VIDEO trial models was 425, and in the OAI study without trajectory group 1, it was 2827 for the left knee, 3049 for the right knee, and 3348 for the generalised model, depending on the missing values of Kellgren–Lawrence grade at baseline.

* The model was constructed using binary logistic regression model and included pain trajectory group (forced entry for the report), vitamin D main effect, and currently working.

† The models was constructed using Cox proportional hazards model, and all 3 included pain trajectory group, age, Centre for Epidemiological Studies Depression score, Kellgren–Lawrence of the examined knee at baseline, use of medications and use of supplements at baseline; in addition to these, left knee model included also sex and comorbidities, right knee model smoking, and the generalised model knee replacement at baseline.

CI, confidence interval; HR, hazard ratio; OAI, Osteoarthritis Initiative; OR, odds ratio.

### 3.3. Descriptors of the pain trajectories

Table 4 contains baseline characteristics of the trajectory groups from both studies.

**Table 4:** Baseline characteristics of the pain trajectory groups.

	The VIDEO trial (N = 474)				The OAI cohort (N = 4796)					
Trajectory group	1	2	3	4	1	2	3	4	5	6
Phenotype	Low-fluctuating	Mild-increasing	Moderate-treatment-sensitive	Severe-treatment-insensitive	None	Low-fluctuating	Mild-increasing	Moderate-treatment-sensitive	Moderate-treatment-sensitive	Severe-treatment-insensitive
	N = 173	N = 188	N = 97	N = 16	N = 1093	N = 1782	N = 1078	N = 163	N = 535	N = 145
Variables	36.4%	39.7%	20.5%	3.4%	22.8%	37.2%	22.5%	3.4%	11.2%	3.0%
Age (median, IQR) Missing (%)	62.0 (58.0-68.0) 0.0	64.0 (58.0-71.0) 0.0	64.0 (59.0-69.0) 0.0	66.0 (61.3-67.8) 0.0	62.0 (53.5-70.0) 5.1	61.0 (53.5-68.0) 5.0	61.0 (54.0-69.0) 5.7	62.0 (55.0-69.0) 2.5	60.0 (53.0-68.8) 11.8	56.0 (51.8-63.0) 15.9
Sex (%)										
Women	59.5	63.3	56.7	75.0	53.1	58.0	58.0	68.1	64.9	74.5
BMI (median, IQR) Missing (%)	27.7 (24.8-30.2) 0.6	28.8 (25.4-32.3) 0.0	30.8 (27.8-35.1) 0.0	32.8 (28.6-38.8) 0.0	26.4 (23.6-29.8) 0.0	27.7 (24.7-31.1) 0.1	29.1 (25.9-32.4) 0.2	29.2 (26.5-32.5) 0.0	30.4 (27.4-34.3) 0.2	32.7 (28.0-37.4) 0.0
Smoking (%)										
Current	3.5	5.3	6.2	18.8	3.6	4.9	5.7	15.3	13.3	20.7
Current-not regular					0.2	0.3	0.3	0.0	0.0	0.0
Former	45.1	45.2	48.5	25.0	36.0	39.6	44.3	41.8	40.9	31.0
Never smoked	49.7	48.9	44.3	56.3	60.2	55.2	49.7	42.9	45.8	48.3
Missing	1.7	0.5	1.0	0.0	0.0	0.0	0.0	0.0	0.0	0.0
Alcohol use (%)										
Yes	83.8	83.5	82.5	81.3	84.2	81.6	80.5	77.3	68.8	57.9
Missing	0.0	0.0	0.0	0.0	0.0	0.0	0.0	0.0	0.0	0.0
Currently working (%)										
Yes	52.6	39.4	28.9	33.3	63.7	64.6	58.5	54.0	56.4	51.0
Missing	0.0	0.0	0.0	6.3	0.0	0.0	0.0	0.0	0.0	0.0
Physical activity (%) sport/hobby>1/month					N/A					
Yes	53.8	48.9	35.1	40.0						
Missing	0.6	1.1	0.0	6.3						
Depression										
Beck Depression Inventory (median, IQR)	1.0 (0.0-2.0)	1.0 (0.0-4.0)	1.0 (1.0-4.0)	2.0 (0.3-4.5)						
Centre for Epidemiological Studies Depression Scale (median, IQR)					3.0 (1.0-6.0)	4.0 (2.0-8.0)	5.0 (2.0-11.0)	7.0 (3.0-12.8)	7.0 (3.0-14.0)	13.0 (5.0-21.0)
Missing (%)	0.0	0.5	0.0	0.0	0.6	1.5	0.9	1.8	2.8	2.8
Quality of Life WHOQoL-Bref (median, IQR)					N/A					
Physical domain	75.0 (64.3-82.1)	64.3 (53.6-75.0)	57.1 (42.9-64.3)	55.4 (32.1-59.8)						
Psychological domain	75.0 (66.7-80.0)	70.8 (62.5-79.2)	70.8 (60.0-79.2)	66.7 (58.3-74.0)						
Social domain	75.0 (66.7-83.3)	75.0 (58.3-83.3)	66.7 (58.3-83.3)	66.7 (52.1-75.0)						
Environmental domain	81.3 (71.9-90.6)	78.1 (68.8-84.4)	75.0 (65.6-84.4)	68.8 (62.5-83.6)						
Missing (%)	1.2	1.1	2.1	0.0						
Comorbidities (%)	N/A									
None					82.2	79.6	74.3	70.5	59.6	53.8
One					10.9	13.5	16.0	17.2	23.0	28.3
More than one					6.9	6.9	9.7	12.3	17.4	17.9
Missing					0.0	0.0	0.0	0.0	0.0	0.0
Medications (%) Use of analgesics, NSAIDs, and steroids										
Yes	47.4	59.0	69.1	86.7	18.8	30.0	48.1	63.8	58.9	72.4
Missing	6.9	1.6	1.0	6.3	0.0	0.0	0.0	0.0	0.0	0.0
Supplements (%) Use of glucosamine and chondroitin										
Yes	36.4	29.3	20.6	6.7	26.6	36.1	39.8	36.2	33.5	15.9
Missing (%)	6.9	1.6	1.0	6.3	0.0	0.0	0.0	0.0	0.0	0.0
Kellgren–Lawrence grade (%) (Index/Worse knee)*										
0	2.3	0.0	2.1	0.0	41.1	28.6	18.7	11.0	12.9	8.3
1	27.7	24.2	22.7	37.5	19.4	16.4	11.3	12.3	7.1	8.3
2	39.9	38.7	34.0	25.0	23.0	30.9	28.8	25.8	31.0	31.0
3	25.4	29.6	35.1	18.8	9.1	15.2	26.3	27.0	27.9	32.4
4	4.6	7.5	5.2	12.5	1.3	4.2	9.6	22.1	10.3	6.9
Missing (%)	0.0	1.1	1.0	6.3	6.2	4.7	5.3	1.8	10.8	13.1
Knee replacement at baseline (%)	N/A									
Left					0.5	0.4	0.4	1.2	1.1	0.0
Right					0.5	0.7	1.0	0.6	1.3	0.7
Knee replacement during follow-up (%)										
Left					0.6	3.3	7.5	31.9	11.4	7.6
Right					0.7	3.8	8.3	30.7	10.7	4.1
Bilateral					0.4	1.4	3.1	19.6	4.3	1.4
Index	1.2	3.7	2.1	12.5						
Contralateral	5.2	4.3	10.3	18.8						
Bilateral	0.6	0.0	1.0	0.0						
Mortality (%)										
Yes	0.6	1.1	1.0	6.3	6.2	5.9	6.3	3.7	9.7	4.1

* Index knee refers to the VIDEO trial, and Worse knee to the OAI study.

BMI, body mass index; IQR, interquartile range; N/A, not applicable due to exclusion criteria; N/A, not applicable or not assessed; NSAID, nonsteroidal anti-inflammatory drug; OAI, Osteoarthritis Initiative; WHOQoL-Bref, The World Health Organization Quality of Life Instrument.

In the VIDEO trial, we created 2 models using the first and fourth trajectory groups as references. The higher BMI and the lower physical domain of WHOQoL-Bref were associated with being in all groups compared to the first group. The higher psychological domain of WHOQoL-Bref was associated with membership of the second and third trajectory groups. The second model aimed to distinguish the third and fourth groups: however, no single analysed variable showed a significant result (Appendix, available as supplemental digital content at http://links.lww.com/PAIN/B88).

In the OAI study, the first, fourth, and sixth groups were of interest, and we created 3 models using each as a reference. Members of any painful group, compared to those with minimal pain, were significantly more likely to be younger women with higher BMI, depression score, KL grade 2 or more, and using painkillers. The membership in the fifth group compared to the fourth group was only negatively significantly associated with KL grade 1. Finally, older age, lower BMI, lower depression score, and use of supplements were significantly associated with the fourth and fifth groups compared to the sixth group. Other variables showed limited potential in distinguishing the pain groups (Appendix, available as supplemental digital content at http://links.lww.com/PAIN/B88).

### 3.4. Sensitivity analyses

Remodelled pain trajectories without mortality cases were the same as in the original model (Appendix, available as supplemental digital content at http://links.lww.com/PAIN/B88). In the OAI study, we identified 6 left and 6 right knee pain trajectories, described by higher-order curves like the original/generalised pain trajectory model. In the dual trajectory model, left knee pain development slightly changed in terms of group percentage and posterior probabilities, given the additional information on right knee pain trajectories. Joint probabilities showed that 64.2 of individuals were classified in the overlapping left and right knee pain groups (Appendix, available as supplemental digital content at http://links.lww.com/PAIN/B88).

## 4. Discussion

We identified knee OA subgroups/phenotypes based on pain trajectories. The number of trajectory groups, their size, and pattern of development differed due to study inclusion criteria, sample size, and follow-up duration of the VIDEO trial and the OAI study. However, due to observable similarities, we identified 4 OA phenotypes from these: low-fluctuating, mild-increasing, moderate-treatment-sensitive, and severe-treatment-insensitive pain. We found that pain and functional limitations in OA measured by WOMAC questionnaire showed the same development over time. Importantly, we identified a phenotype with severe pain that did not benefit from analgesics and had the same chance for knee replacement as the low-fluctuating phenotype. We also identified a subgroup most likely to benefit from knee replacement. Finally, using baseline factors, we were able to distinguish painful from minimally painful groups but found little to differentiate moderate from severe pain groups.

We used 2 high-quality studies of different design, size, and follow-up duration to overcome some of their complementary drawbacks. Unlike some previous studies, ours did not use any method to additionally select/match participants or make the 2 studies more similar.^6,26^ Instead, we relied on a method that selected latent classes for dealing with heterogeneity.^23^ In group-based modelling, we permitted small groups to be detected when the model fit supported it. We also managed to replicate the small-sized groups, reducing the chance of spurious classes' detection. Although studies had different intervals of the outcome assessment, it provided additional evidence of the consistency and robustness of the findings. Both studies involved OA patients at different disease stages. The baseline in each case was related to the study; it is not the disease baseline because there is still a lack of OA onset definition in the field overall.^19^ However, our studies had different follow-up durations, allowing us to observe time-effects more comprehensively. However, due to different follow-up durations, we did not directly compare trajectory groups between the samples. Although different inclusion and exclusion criteria were used to some extent, samples were similar in terms of demographic and lifestyle factors. As expected, trial participants were more severe in clinical and radiographic aspects. We used a set of baseline characteristics that did not entirely overlap. Although this was a study limitation, it represented the diversity of OA covariate measures used.

There were 4 phenotypes identified in the trial and replicated in the OAI study. We named phenotypes by indicating baseline pain and its pattern or responsiveness to treatments. The minimal pain trajectory group from the OAI study represented people with minimal-to-neglected knee pain, thus not assumed an OA phenotype. These were sampled in the cohort but not the trial. The first trajectory group selected in the VIDEO trial and the second trajectory group in the OAI study presented the low-fluctuating phenotype. In the short term, it showed pain improvement but fluctuated in the long term, albeit staying quite low. This phenotype included slightly more than a third of both samples and reflected reports from previous studies.^3,6,26,27^ The second trajectory group in the trial and the third in the cohort represented the second mild-increasing phenotype (also reported before).^6,26^ This phenotype is the only more common one in the trial than in the cohort. The remarkable observation was related to the third moderate-treatment-sensitive phenotype: third trajectory group in the trial and the fourth and fifth groups in the cohort. In a 3-year window, this phenotype presented moderate-increasing pain in both studies. However, in longer term, this phenotype divided into 2 subgroups: in one, patients benefited from knee replacement (fourth trajectory), whereas in another, patients continued to experience moderate pain despite significant analgesic effects (fifth trajectory). This phenotype included 15% to 20% of the samples. Similar observations were found in studies using the CAS-K^26^ and CHECK^3^ cohorts, but not in the 5-trajectory model previously identified in the OAI study.^6,26^ However, previous studies did not examine treatment effects on pain trajectories. Finally, the fourth phenotype included 3% of both our samples. It was also shown earlier^3,6,26^ and here additionally described by severe-treatment-insensitive pain (fourth group in the trial and sixth group in the cohort).

Furthermore, we found that functional limitations followed identical development to pain, indicating that people experienced these 2 outcomes very similarly or could not distinguish between them. Some studies examined functional limitation in knee OA,^11,32^ but none of these did not look into interplay between pain and functional limitation over time. Although the first 3 phenotypes were responsive to currently available analgesics, the fourth was not. OA phenotypes were not significantly associated with knee replacement during a 3-year follow-up because only a few replacements took place during the period. However, in the longer term—as the number of replacements increased—mild-increasing and moderate-treatment-sensitive phenotypes but not severe-treatment-insensitive had significant odds of having knee replacement when compared to the mild-fluctuating one. The severe-treatment-insensitive phenotype in the OAI study included the youngest women with the highest BMI and depression score, with more comorbidities, using analgesics, but without pain relief. The left and right knee trajectories and their dual model showed that most people develop the same pain pattern irrespective of laterality, probably due to central pain processing. The overlapping group percentage was likely lower due to individuals' unilateral pathologies.

The baseline clinical and lifestyle factors in our study were modest in differentiating the phenotypes. Overall, the variable with a consistently positive relationship to pain seemed to be BMI, indicating metabolic differences between phenotypes. The age effect was transposed between study samples. As previously discussed, the exclusion criteria in the OAI study likely led to a healthier population being selected.^6^ More generally, age effect can be a random observation unrelated to pain phenotypes, instead indicating time-dependent exposure and a molecular process to be detected. Interestingly, 75% of the fourth phenotype were women, indicating a sex-specific mechanism of severe-treatment-insensitive pain. Finally, the 9-year follow-up is the longest regular/annual follow-up so far in knee OA, and it has given us better insights into long-term pain, the slow-progressing character of OA, and its relation to OA end-stage.

To conclude, our approach provided robust results regarding pain experience for OA patient phenotyping with clinical, research, and trial-design relevance. Pain should remain the primary outcome under investigation because functional limitations do not add information. Besides pain duration, we should also consider pain intensity. The cutoff for inclusion in clinical trials should be pain intensity above 20%, and for the sensitivity analyses above 50% of the scale. This range is also the indicator for delivery of currently available treatments. Patients experiencing pain above 50% of the scale need novel pharmacological treatments and careful consideration of safety issues due to comorbidities. Due to the reproducibility between study designs, it creates a template for reanalysing available longitudinal data pools with further characterisation. To improve phenotype differentiation beyond this report, we suggest using molecular and genetic tools^9^ that should provide inside into dysregulated molecular pathways to target. Then, pain with additional tools will lead to an optimal set of criteria for selecting patients for treatment options and future OA clinical trials.

## Conflict of interest statement

All authors have completed the ICMJE uniform disclosure form and declare: M.R. Radojčić, N.K. Arden, and F. Birrell receive research grants from the Versus Arthritis and X. Yang from the China Scholarship Council that supported the submitted work; N.K. Arden receives grants and personal fees from Merck, Flexion, Regeneron, Pfizer, and Lilly, and C. Cooper receives personal fees from Amgen, Danone, Eli Lilly, GSK, Kyowa Kirin, Medtronic, Merck, Nestle, Novartis, Pfizer, Roche, Servier, Shire, Takeda, and UCB outside the submitted work; no intellectual property broadly relevant to the submitted work; no other relationships or activities that could seem to have influenced the submitted work.

## Appendix A. Supplemental digital content

Supplemental digital content associated with this article can be found online at http://links.lww.com/PAIN/B88.

## References

[1] Andruff H Carraro N Thompson A Gaudreau P Latent class growth modelling: a tutorial. Tutorials Quantitative Methods Psychol 2009;5:11–24.

[2] Arden NK Cro S Sheard S Dore CJ Bara A Tebbs SA Hunter DJ James S Cooper C O'Neill TW Macgregor A Birrell F Keen R The effect of vitamin D supplementation on knee osteoarthritis, the VIDEO study: a randomised controlled trial. Osteoarthritis Cartilage 2016;24:1858–66. 27264058 10.1016/j.joca.2016.05.020 PMC5045720

[3] Bastick AN Wesseling J Damen J Verkleij SP Emans PJ Bindels PJ Bierma-Zeinstra SM Defining knee pain trajectories in early symptomatic knee osteoarthritis in primary care: 5-year results from a nationwide prospective cohort study (CHECK). Br J Gen Pract 2016;66:e32–e39. 26639946 10.3399/bjgp15X688129 PMC4684033

[4] Beck AT Steer RA Ball R Ranieri W Comparison of Beck depression Inventories -IA and -II in psychiatric outpatients. J Pers Assess 1996;67:588–97. 8991972 10.1207/s15327752jpa6703_13

[5] Bellamy N WOMAC: a 20-year experiential review of a patient-centered self-reported health status questionnaire. J Rheumatol 2002;29:2473–6. 12465137

[6] Collins JE Katz JN Dervan EE Losina E Trajectories and risk profiles of pain in persons with radiographic, symptomatic knee osteoarthritis: data from the osteoarthritis initiative. Osteoarthritis Cartilage 2014;22:622–30. 24662734 10.1016/j.joca.2014.03.009 PMC4028704

[7] Cross M Smith E Hoy D Nolte S Ackerman I Fransen M Bridgett L Williams S Guillemin F Hill CL Laslett LL Jones G Cicuttini F Osborne R Vos T Buchbinder R Woolf A March L The global burden of hip and knee osteoarthritis: estimates from the global burden of disease 2010 study. Ann Rheum Dis 2014;73:1323–30. 24553908 10.1136/annrheumdis-2013-204763

[8] Deveza LA Melo L Yamato TP Mills K Ravi V Hunter DJ Knee osteoarthritis phenotypes and their relevance for outcomes: a systematic review. Osteoarthritis Cartilage 2017;25:1926–41. 28847624 10.1016/j.joca.2017.08.009

[9] Felson DT Identifying different osteoarthritis phenotypes through epidemiology. Osteoarthritis Cartilage 2010;18:601–4. 20175975 10.1016/j.joca.2010.01.007 PMC3474706

[10] Harper A Power M Grp W Development of the World health Organization WHOQOL-BREF quality of life assessment. Psychol Med 1998;28:551–8. 9626712 10.1017/s0033291798006667

[11] Holla JF van der Leeden M Heymans MW Roorda LD Bierma-Zeinstra SM Boers M Lems WF Steultjens MP Dekker J Three trajectories of activity limitations in early symptomatic knee osteoarthritis: a 5-year follow-up study. Ann Rheum Dis 2014;73:1369–75. 23716068 10.1136/annrheumdis-2012-202984

[12] Hunter DJ Bierma-Zeinstra S Osteoarthritis. Lancet 2019;393:1745–59. 31034380 10.1016/S0140-6736(19)30417-9

[13] Hunter DJ Schofield D Callander E The individual and socioeconomic impact of osteoarthritis. Nat Rev Rheumatol 2014;10:437–41. 24662640 10.1038/nrrheum.2014.44

[14] Jones BL Nagin DS Advances in group-based trajectory modeling and an SAS procedure for estimating them. Sociol Method Res 2007;35:542–71.

[15] Jones BL Nagin DS Roeder K A SAS procedure based on mixture models for estimating developmental trajectories. Sociol Method Res 2001;29:374–93.

[16] Jung T Wickrama K An introduction to latent class growth analysis and growth mixture modeling. Social Personal Psychol Compass 2008;2:302–17.

[17] Karsdal MA Christiansen C Ladel C Henriksen K Kraus VB Bay-Jensen AC Osteoarthritis—a case for personalized health care? Osteoarthritis Cartilage 2014;22:7–16. 24216058 10.1016/j.joca.2013.10.018

[18] Kellgren JH Lawrence JS Radiological assessment of osteo-arthrosis. Ann Rheum Dis 1957;16:494–502. 13498604 10.1136/ard.16.4.494 PMC1006995

[19] Losina E Collins JE Forecasting the future pain in hip OA: can we rely on pain trajectories? Osteoarthritis Cartilage 2016;24:765–7. 26854793 10.1016/j.joca.2016.01.989

[20] Mauish ME The use of psychological testing for treatment planning and outcomes assessment: instruments for adults. Mahwah: Lawrence Eribaum Associates, 2004.

[21] Mobasheri A Saarakkala S Finnila M Karsdal MA Bay-Jensen AC van Spil WE Recent advances in understanding the phenotypes of osteoarthritis. F1000Res 2019;8:F1000 Faculty Rev-2091. 10.12688/f1000research.20575.1 PMC6913225 31885861

[22] Murray CJ Vos T Lozano R Naghavi M Flaxman AD Michaud C Ezzati M Shibuya K Salomon JA Abdalla S Aboyans V Abraham J Ackerman I Aggarwal R Ahn SY Ali MK Alvarado M Anderson HR Anderson LM Andrews KG Atkinson C Baddour LM Bahalim AN Barker-Collo S Barrero LH Bartels DH Basanez MG Baxter A Bell ML Benjamin EJ Bennett D Bernabe E Bhalla K Bhandari B Bikbov B Bin Abdulhak A Birbeck G Black JA Blencowe H Blore JD Blyth F Bolliger I Bonaventure A Boufous S Bourne R Boussinesq M Braithwaite T Brayne C Bridgett L Brooker S Brooks P Brugha TS Bryan-Hancock C Bucello C Buchbinder R Buckle G Budke CM Burch M Burney P Burstein R Calabria B Campbell B Canter CE Carabin H Carapetis J Carmona L Cella C Charlson F Chen H Cheng AT Chou D Chugh SS Coffeng LE Colan SD Colquhoun S Colson KE Condon J Connor MD Cooper LT Corriere M Cortinovis M de Vaccaro KC Couser W Cowie BC Criqui MH Cross M Dabhadkar KC Dahiya M Dahodwala N Damsere-Derry J Danaei G Davis A De Leo D Degenhardt L Dellavalle R Delossantos A Denenberg J Derrett S Des Jarlais DC Dharmaratne SD Dherani M Diaz-Torne C Dolk H Dorsey ER Driscoll T Duber H Ebel B Edmond K Elbaz A Ali SE Erskine H Erwin PJ Espindola P Ewoigbokhan SE Farzadfar F Feigin V Felson DT Ferrari A Ferri CP Fevre EM Finucane MM Flaxman S Flood L Foreman K Forouzanfar MH Fowkes FG Fransen M Freeman MK Gabbe BJ Gabriel SE Gakidou E Ganatra HA Garcia B Gaspari F Gillum RF Gmel G Gonzalez-Medina D Gosselin R Grainger R Grant B Groeger J Guillemin F Gunnell D Gupta R Haagsma J Hagan H Halasa YA Hall W Haring D Haro JM Harrison JE Havmoeller R Hay RJ Higashi H Hill C Hoen B Hoffman H Hotez PJ Hoy D Huang JJ Ibeanusi SE Jacobsen KH James SL Jarvis D Jasrasaria R Jayaraman S Johns N Jonas JB Karthikeyan G Kassebaum N Kawakami N Keren A Khoo JP King CH Knowlton LM Kobusingye O Koranteng A Krishnamurthi R Laden F Lalloo R Laslett LL Lathlean T Leasher JL Lee YY Leigh J Levinson D Lim SS Limb E Lin JK Lipnick M Lipshultz SE Liu W Loane M Ohno SL Lyons R Mabweijano J MacIntyre MF Malekzadeh R Mallinger L Manivannan S Marcenes W March L Margolis DJ Marks GB Marks R Matsumori A Matzopoulos R Mayosi BM McAnulty JH McDermott MM McGill N McGrath J Medina-Mora ME Meltzer M Mensah GA Merriman TR Meyer AC Miglioli V Miller M Miller TR Mitchell PB Mock C Mocumbi AO Moffitt TE Mokdad AA Monasta L Montico M Moradi-Lakeh M Moran A Morawska L Mori R Murdoch ME Mwaniki MK Naidoo K Nair MN Naldi L Narayan KM Nelson PK Nelson RG Nevitt MC Newton CR Nolte S Norman P Norman R O'Donnell M O'Hanlon S Olives C Omer SB Ortblad K Osborne R Ozgediz D Page A Pahari B Pandian JD Rivero AP Patten SB Pearce N Padilla RP Perez-Ruiz F Perico N Pesudovs K Phillips D Phillips MR Pierce K Pion S Polanczyk GV Polinder S Pope CA III Popova S Porrini E Pourmalek F Prince M Pullan RL Ramaiah KD Ranganathan D Razavi H Regan M Rehm JT Rein DB Remuzzi G Richardson K Rivara FP Roberts T Robinson C De Leon FR Ronfani L Room R Rosenfeld LC Rushton L Sacco RL Saha S Sampson U Sanchez-Riera L Sanman E Schwebel DC Scott JG Segui-Gomez M Shahraz S Shepard DS Shin H Shivakoti R Singh D Singh GM Singh JA Singleton J Sleet DA Sliwa K Smith E Smith JL Stapelberg NJ Steer A Steiner T Stolk WA Stovner LJ Sudfeld C Syed S Tamburlini G Tavakkoli M Taylor HR Taylor JA Taylor WJ Thomas B Thomson WM Thurston GD Tleyjeh IM Tonelli M Towbin JA Truelsen T Tsilimbaris MK Ubeda C Undurraga EA van der Werf MJ van Os J Vavilala MS Venketasubramanian N Wang M Wang W Watt K Weatherall DJ Weinstock MA Weintraub R Weisskopf MG Weissman MM White RA Whiteford H Wiebe N Wiersma ST Wilkinson JD Williams HC Williams SR Witt E Wolfe F Woolf AD Wulf S Yeh PH Zaidi AK Zheng ZJ Zonies D Lopez AD AlMazroa MA Memish ZA Disability-adjusted life years (DALYs) for 291 diseases and injuries in 21 regions, 1990-2010: a systematic analysis for the Global Burden of Disease Study 2010. Lancet 2012;380:2197–223. 23245608 10.1016/S0140-6736(12)61689-4

[23] Nagin DS Group-based modeling of development. Cambridge: Harvard University Press, 2005.

[24] Nagin DS Odgers CL Group-based trajectory modeling in clinical research. Annu Rev Clin Psychol 2010;6:109–38. 20192788 10.1146/annurev.clinpsy.121208.131413

[25] Nagin DS Tremblay RE Analyzing developmental trajectories of distinct but related behaviors: a group-based method. Psychol Methods 2001;6:18–34. 11285809 10.1037/1082-989x.6.1.18

[26] Nicholls E Thomas E van der Windt DA Croft PR Peat G Pain trajectory groups in persons with, or at high risk of, knee osteoarthritis: findings from the Knee Clinical Assessment Study and the Osteoarthritis Initiative. Osteoarthritis Cartilage 2014;22:2041–50. 25305072 10.1016/j.joca.2014.09.026 PMC4256061

[27] Pan F Tian J Aitken D Cicuttini F Jones G Predictors of pain severity trajectory in older adults: a 10.7-year follow-up study. Osteoarthr Cartilage 2018;26:1619–26. 10.1016/j.joca.2018.08.002 30121348

[28] Roubille C Pelletier JP Martel-Pelletier J New and emerging treatments for osteoarthritis management: will the dream come true with personalized medicine? Expert Opin Pharmacother 2013;14:2059–77. 24044485 10.1517/14656566.2013.825606

[29] Sharma L Kapoor D Issa S Epidemiology of osteoarthritis: an update. Curr Opin Rheumatol 2006;18:147–56. 16462520 10.1097/01.bor.0000209426.84775.f8

[30] Van Spil WE Kubassova O Boesen M Bay-Jensen AC Mobasheri A Osteoarthritis phenotypes and novel therapeutic targets. Biochem Pharmacol 2019;165:41–8. 30831073 10.1016/j.bcp.2019.02.037

[31] Walsh DA Stocks J New therapeutic targets for osteoarthritis pain. SLAS Discov 2017;22:931–49. 28683214 10.1177/2472555217716912

[32] White DK Neogi T Nguyen US Niu J Zhang Y Trajectories of functional decline in knee osteoarthritis: the Osteoarthritis Initiative. Rheumatology (Oxford) 2016;55:801–8. 26705330 10.1093/rheumatology/kev419 PMC5009418

